# Profiles of Depressive Symptoms Among Men Who Have Sex With Men and Transgender Women During the COVID-19 Outbreak in Mexico: A Latent Class Analysis

**DOI:** 10.3389/fpubh.2021.598921

**Published:** 2021-06-07

**Authors:** Diego Cerecero-Garcia, Heleen Vermandere, Ietza Bojorquez, José Gómez-Castro, José Arturo Sánchez-Ochoa, Araczy Martínez-Dávalos, Ivonne Huerta-Icelo, Sergio Bautista-Arredondo

**Affiliations:** ^1^Center for Health Systems Research, National Institute of Public Health, Cuernavaca, Mexico; ^2^Department of Population Studies, El Colegio de la Frontera Norte, Tijuana, Mexico

**Keywords:** COVID-19, mental health, depressive symptoms, Mexico, men who have sex with men

## Abstract

The impact of the COVID-19 outbreak on mental health among HIV high-risk populations is not known. We assess the prevalence of depressive symptoms (DS) and explore the association with characteristics related to the COVID-19 pandemic. We conducted an online survey among 881 men who have sex with men (MSM) and transgender women (TGW) assessing the presence of DS using the Center for Epidemiological Studies Depression Scale (CESD-10); results were compared with previously self-reported DS and national data. We applied latent class analysis (LCA) to identify classes of participants with similar COVID-19 related characteristics. The overall prevalence of significant DS was 53.3%. By LCA posterior probabilities we identified three classes: (1) minimal impact of COVID-19 (54.1%), (2) objective risk for COVID-19 (41.5%), and (3) anxiety and economic stress caused by COVID-19 (4.4%). Multivariate logistic regression showed that compared with those in class one, the odds to have significant DS were almost five times higher for those in class three. Our findings suggest high levels of depression among MSM and TGW in Mexico during the COVID-19 pandemic and highlight the need for the provision of targeted psychological interventions to minimize the impacts of COVID-19 on the mental health.

## Introduction

On January 30, 2020, the World Health Organization (WHO) declared a public health emergency after identifying a cluster of unexplained cases of pneumonia in Wuhan, China, subsequently identified as COVID-19, caused by the novel coronavirus SARS-CoV-2 (1). In Mexico, as in the rest of the world, the spread of confirmed cases and deaths has increased dramatically, reaching 2,187,910 and 197,219, respectively, by March 21, 2021 (2). Since the beginning of the epidemic, the Mexican Ministry of Health (MoH) has implemented a series of preventive measures including restricted mobility, sanitation and hygiene, and social distancing (including school closures, suspension of non-essential work activities, cancellation of massive events, and close of restaurants and bars).

The COVID-19 pandemic and the uncertainty it creates in all aspects of life threaten people's physical and mental health. Elevated rates of anxiety, depression, posttraumatic stress disorder (PTSD), and harmful social behaviors (3) have been reported recently (4). A study carried out in 194 cities in China during the COVID-19 outbreak found that 54% of respondents rated the psychological impact of the COVID-19 outbreak as moderate or severe. The prevalence of mild anxiety and depression among this population was 29 and 17%, respectively (5). Another poll of 5,000 Chinese citizens showed PTSD symptoms among 21.5% of the participants, which resembles the prevalence of PTSD (28.9%) and depression (31.2%) experienced by quarantined citizens during the 2003 SARS outbreak (6). In the US, about a third of adults (32%) reported in March 2020 that worry and stress related to COVID-19 harmed their mental health, 14% reported a more significant impact (7). Also, a recent literature review exploring the COVID-19 pandemic consequences on mental health revealed lower psychological well-being and higher scores of anxiety and depression in the general population, compared to before COVID-19 (8).

Evidence on the association between COVID-19 and mental health suggests that protective factors for stress, anxiety, and depression, include a high level of confidence in doctors, perceived survival likelihood, and low risk of contracting COVID-19 (9). Simultaneously, extreme fear, sleep deprivation, and living in severely afflicted areas are significant risk factors for COVID-19-related psychological distress (10). However, the available studies have been conducted mainly in China and other countries initially affected by the pandemic (7–11). The evidence remains limited in Latin American countries, where the current epicenter of the pandemic is, according to WHO. Furthermore, there is a severe lack of knowledge about the impact of COVID-19 on the mental health of highly vulnerable populations such as men who have sex with men (MSM) and transgender women (TGW).

In this paper, we used an online questionnaire to assess the prevalence of depressive symptoms (DS) among a sample of MSM and TGW during the 1st month of the COVID-19 outbreak in Mexico. We also document the association of DS to specific aspects linked to the COVID-19 pandemic, such as loss of a job, perceived risk of acquiring COVID-19, and change in sexual behavior due to COVID-19.

## Methods

### Study Overview and Population

Between April 20th and 27th, 2020, we conducted an online survey about COVID-19 knowledge and attitudes among MSM and TGW. We sent an e-mail invitation to participants in an HIV pre-exposure prophylaxis (PrEP) demonstration project (ImPrEP) with more than 2,000 individuals recruited in three cities of Mexico: Mexico City, Guadalajara, and Puerto Vallarta. We also sent the e-mail invitation to 600 MSM and TGW not enrolled in ImPrEP but contacted as part of the study's demand generation activities in venues frequently used by these populations to meet potential sex partners. Participants provided consent and contact information to be invited to participate in other studies. The survey instrument included questions on demographic characteristics, social support, general knowledge of COVID-19, the preventive measures to reduce the risk of contagion, risk factors and symptoms of COVID-19, the pandemic's impact on employment, and mental health indicators. Participants provided informed consent. The Institutional Review Board (IRB) of the National Institute of Public Health of Mexico reviewed and authorized the survey (IRB number: CI-241-2020).

### Measures

#### Depressive Symptoms

We used the 10-item version of the Center for Epidemiological Studies Depression Scale (CESD-10) to assess the presence of DS among survey participants. The CESD-10 measures the frequency of DS experienced in the past week, including a sense of loneliness, fear, depression, sleep disturbances, and feelings of helplessness and hopelessness (12). Ratings are based on a four-point response format from 0 (rarely or never) to 3 (mostly or always). Total scores range from 0 to 30, and higher scores imply higher levels of DS. The CESD- 10 has demonstrated good internal reliability in the general population and has been used in past studies to assess DS among MSM and TGW PrEP users (13, 14) and the general Mexican population (15, 16). The CESD-10 provides an estimate of DS prevalence within a study population rather than a clinical diagnosis of depression. A cut-off score of ten or higher indicates the presence of significant DS (12).

We had access to data on DS before the onset of the COVID-19 pandemic for ImPrEP participants who answered the SF-36-questionnaire in 2019 (*n* = 108). We were able to compare the mental well-being before and during the outbreak for this subsample of individuals. The mental health module of the SF-36 includes five questions about the frequency of depressive symptoms experienced in the past 4 weeks. Items include indicators of psychological distress and well-being. Ratings are based on a 6-point response format from 1 (all of the time) to 6 (never). Scores are calculated as the sum of every item (range from 5 to 30), and higher scores represent better health status. Raw scores are then transformed to a scale from 0 to 100. We used a cut-off of 50 or lower in the SF-36 score to indicate the presence of significant DS (17).

To include an additional reference measure of DS, we also analyzed data from the 2018 wave of the National Survey of Health and Nutrition (ENSANUT 2018). The ENSANUT 2018 is a probabilistic representative sample of the Mexican population in all 32 states of Mexico. The ENSANUT 2018 assessed the presence of DS among respondents with the CESD-7, which is a shortened version of the CESD, which includes seven items whose ability to differentiate people with significant DS has been previously documented in the Mexican population (18). Additional details on the methodological design of the ENSANUT 2018 are reported elsewhere (19).

#### Demographics

We collected demographic characteristics of participants at the time of the survey, such as educational level (university degree or higher vs. less than university), age, gender, and state of residence (those who live in Mexico City vs. those living in other states of Mexico). We also assessed the perceived level of social support (having family or friends who can lend money if necessary and having family or friends who can take them to the hospital if sick). We created a categorical variable that indicates no social support (=0), at least one type of social support (=1), or both types of support (=2).

#### Covid-19 Related Variables

We explored the effect and risk factors for COVID-19 in different aspects: loss of job because COVID-19 (yes/no), perceived risk of acquiring COVID-19 (not at risk/some risk/high risk), having at least one diagnosed risk factor for severe COVID-19 disease (hypertension, asthma, diabetes, obesity, tuberculosis, or cancer), and potential close contact with someone who has been tested positive for COVID-19 (yes/no). Because of high sexual activity reported during ImPrEP follow-up visits, we explored whether participants reduced their number of sexual partners due to COVID-19 (yes/no). We also explored the number and type of sexual partners (steady/occasional/transactional) in the last 2 weeks, and the use of dating apps.

### Analytical Approach

We explored the distribution of demographics, social support, job loss, and risk of acquiring COVID-19 among those with and without significant DS. We assessed statistical differences using Chi-squared tests. We also compared the prevalence of DS in our sample with that in Mexico's general population (19).

We used latent class analysis (LCA) to identify the number of patterns (i.e., classes) of COVID-19 related variables. LCA is a person-centered approach for identifying common experiences across individuals, in contrast with variable-centered approaches that identify common relationships among variables (20). Compared to other methodologies, LCA offers the opportunity to assess specific combinations of multiple risk factors simultaneously. It uses observed variables to identify common combinations or patterns of experiences that represent population subtypes.

We fitted multiple LCA models using the COVID-19 related variables previously described. We identified latent classes by estimating models with two to seven classes. We selected the best-fitting model based on account fit indices, including the Bayesian Information Criterion (BIC) and the Akaike Information Criterion (AIC) (20). We then estimated posterior probabilities and assigned individuals to the class for which the posterior probability was the highest (i.e., for which they had the closest fit). Following class identification, we estimated logistic regression models to assess the association between the presence of CESD-10 DS and the class assigned to each individual controlling for sociodemographic characteristics.

DS indicate psychological distress that might amount to a full-blown depressive disorder. At the same time, a certain level of DS can be a normal response to stressful circumstances. Thus, the association between DS and COVID-19-related variables might be more robust for DS scores in the middle range, while extremely high scores could indicate psychopathology beyond the stress-related response. To assess whether the association between DS and COVID-19 related variables could be different in different points of the distribution of the CESD-10 score, we used quantile regressions and estimated coefficients at the mean, and Q25, Q50, Q75, and Q90. All the analyses were performed in Stata 15 (21).

## Results

We invited 3,049 individuals to answer the online survey. The response rate was 29% (881 participants), and 595 MSM or TGW completed the online survey (67% of respondents). Seventy-seven percent of completed surveys corresponded to enrollees in the ImPrEP study. The overall prevalence of DS was 53%, 83% reported schooling levels of university or higher, 53% were 28–36 years old, 72% of the respondents lived in Mexico City, and 3% were TGW.

We compared the demographic characteristics of the ImPrEP participants with those not enrolled in ImPrEP. The only statistical difference we found was a higher proportion of enrolees living in Mexico City (Supplementary Table 1).

**Figure 1:**
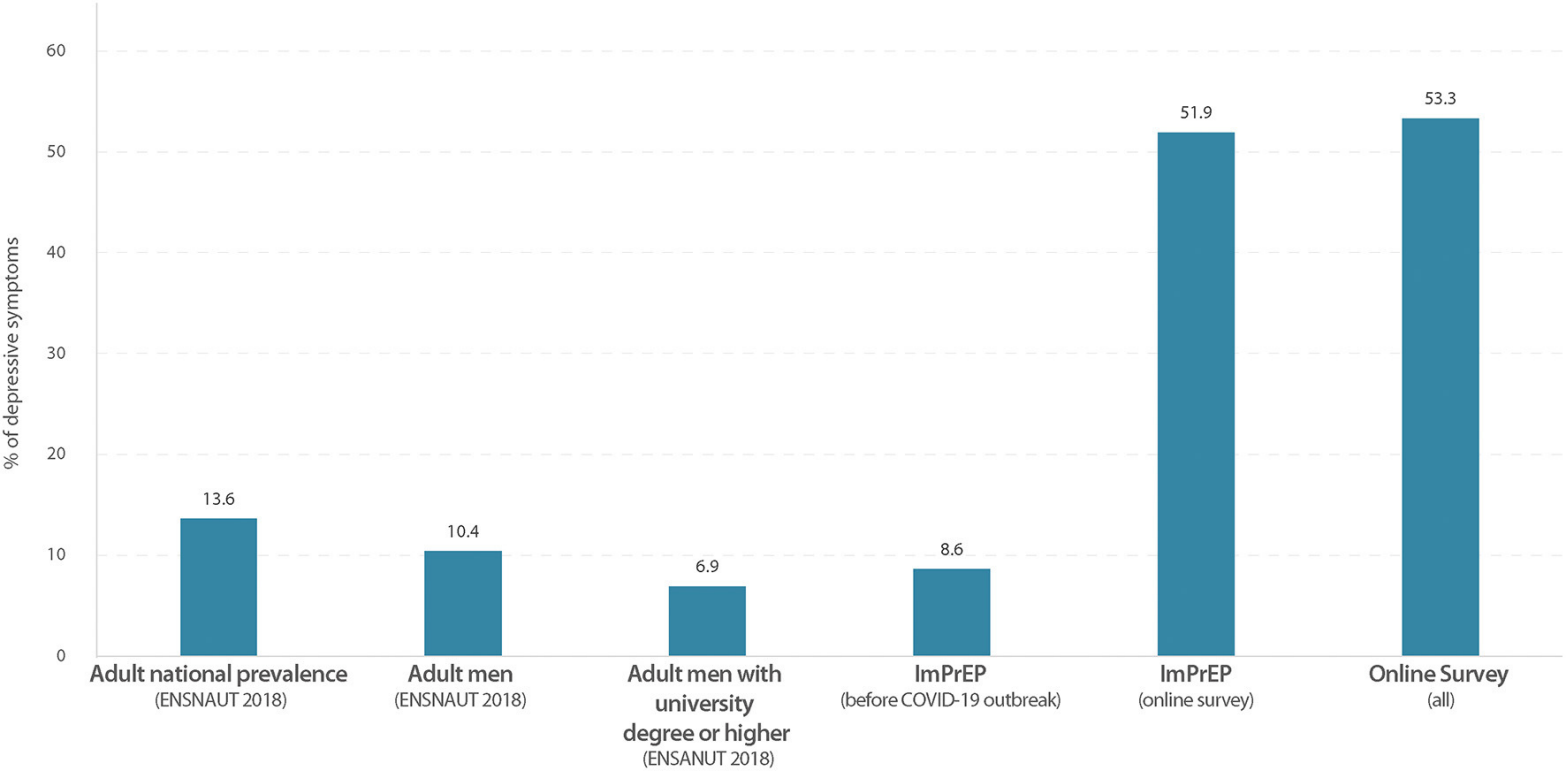
Prevalence of depressive symptoms among online survey participants compared with Mexico's national prevalence.

In Figure 1, we compare the prevalence of significant DS among study participants with Mexico's national prevalence and men's national prevalence, according to ENSANUT 2018. The prevalence in the Mexican adult population was 13.6%, 10.4% among men, and 6.9% among men with a university degree or higher. Significant DS prevalence during the COVID-19 lockdown was six times higher among those enrolled in the ImPrEP study than before the pandemic (51.9 vs. 8.6%).

We explored differences in the distribution of demographic and COVID-19-related variables among participants, stratifying by the presence of significant DS (Table 1). We observed higher rates of DS among the youngest (36 years or younger) and Mexico City dwellers. Among participants without social support, 57.6% had significant DS, compared with 50.3% in the group with high social support. The prevalence of significant DS was 61.3% among participants who lost their jobs because of COVID-19, compared to 50.8% if they did not lose their job. Significant DS were also more frequent among those who considered themselves at high risk of acquiring COVID-19 (67.1%), with at least one medical risk factor for severe stages of COVID-19 (61.2%), and those who know someone diagnosed with COVID-19 (63.2%).

**Table 1:** Demographic characteristics of participants with CESD-10 significant depressive symptoms (*n* = 317) and without significant depressive symptoms (*n* = 278).

**Variable**	**No DS**	**DS**	**Total**	***P-*value**
***n* (%)**	**278 (46.7)**	**317 (53.3)**	**595 (100.0)**	
**DEMOGRAPHICS**				
**Education**				
Less than university	39 (38.2)	63 (61.8)	102 (17.1)	0.059
University degree of higher	239 (48.5)	254 (51.5)	493 (82.9)	
**Age**				
18–27 years	54 (36.0)	96 (64.0)	150 (25.2)	0.002
28–36 years	150 (47.6)	165 (52.4)	315 (52.9)	
>36 years	74 (56.9)	56 (43.1)	130 (21.8)	
**State of residence**				
Lives in Mexico City	186 (43.1)	246 (56.9)	432 (72.6)	0.004
Lives in other state of Mexico	92 (56.4)	71 (43.6)	163 (27.4)	
**Social support**				
Null social support	22 (32.4)	46 (57.6)	68 (11.4)	0.030
Some social support	72 (45.9)	85 (54.1)	157 (26.4)	
High social support	184 (49.7)	186 (50.3)	370 (62.2)	
**Gender**				
Male	273 (47.2)	305 (52.7)	578 (97.1)	0.147
Transgender women	5 (29.4)	12 (70.6)	17 (2.9)	
**Enrolled in ImPrEP study**				
Enrolled	234 (48.2)	252 (51.9)	486 (81.7)	0.141
Not enrolled	44 (40.4)	65 (59.6)	109 (18.3)	
**COVID-19 RELATED VARIABLES**				
**Lost job because of COVID-19**				
Yes	55 (38.7)	87 (61.3)	142 (23.9)	0.029
No	223 (49.2)	230 (50.8)	453 (76.1)	
**Perceived risk of acquiring COVID-19**				
Null risk	46 (59.7)	31 (40.3)	77 (12.9)	<0.001
Some risk	179 (50.1)	178 (49.9)	357 (60.0)	
High risk	53 (32.9)	108 (67.1)	161 (27.1)	
**Has at least one medical risk factor for severe COVID-19 disease**				
Yes	99 (38.8)	156 (61.2)	255 (42.9)	0.001
No	179 (52.6)	161 (47.4)	340 (57.1)	
**Knows someone with COVID-19**				
Yes	63 (36.8)	108 (63.2)	171 (28.7)	0.002
No	215 (50.7)	209 (49.3)	424 (71.3)	
**Had contact with someone with COVID-19**				
Yes	7 (41.2)	10 (58.8)	17 (2.86)	0.642
No	271 (46.9)	307 (53.1)	578 (97.1)	
**Decreased sexual partners because COVID-19**				
Yes	208 (46.4)	240 (53.6)	448 (75.3)	0.802
No	70 (47.6)	77 (52.4)	147 (24.7)	

### Latent Class Analysis

Following the LCA estimation, we summarized our data using a three-class model (Supplementary Table 5). Based on the item-specific response probabilities, latent class labels were:

1. Minimal impact of COVID-19−54%,
2. Objective risk of COVID-19−41%, and
3. Anxiety and economic stress caused by COVID-19−4%.

**Figure 2:**
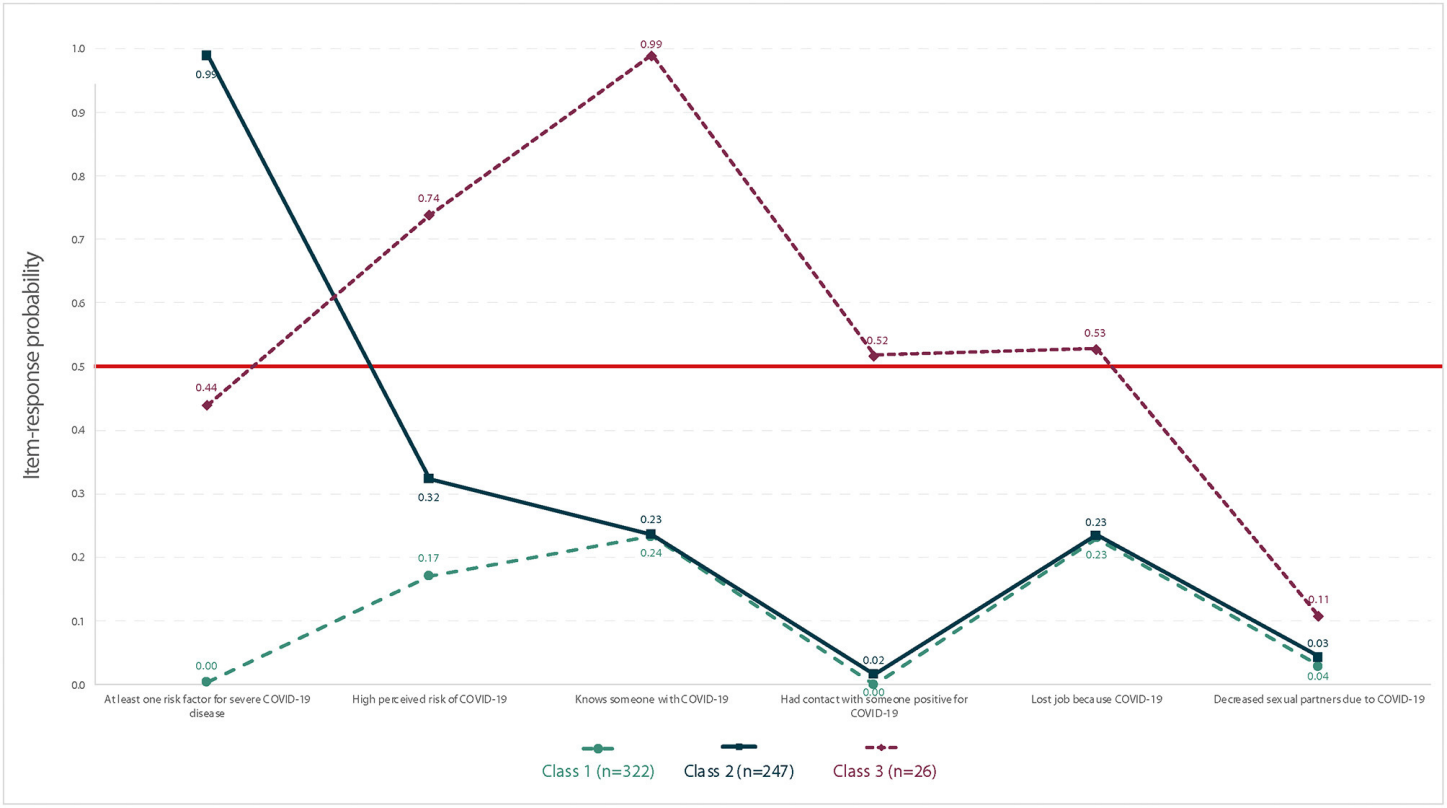
Latent class analysis item-response for the three-class model of MSM and TGW (*n* = 595).

Figure 2 displays the results of the latent class analysis. The attribute of class one is a minimal impact of the pandemic—defined by <25% probability of experience in any indicator of risk or negative effect of COVID-19. Class two is characterized by those objectively at risk for COVID-19 severe disease, with 99% of participants in this group reporting at least one objectively measured risk factor. Class three's feature is anxiety and economic stress caused by COVID-19. It includes participants who lost their jobs due to COVID-19 (53%), knew someone with COVID-19 (99%), perceived high risk of acquiring COVID-19 (74%) or had contact with someone diagnosed with COVID-19 (52%).

**Figure 3:**
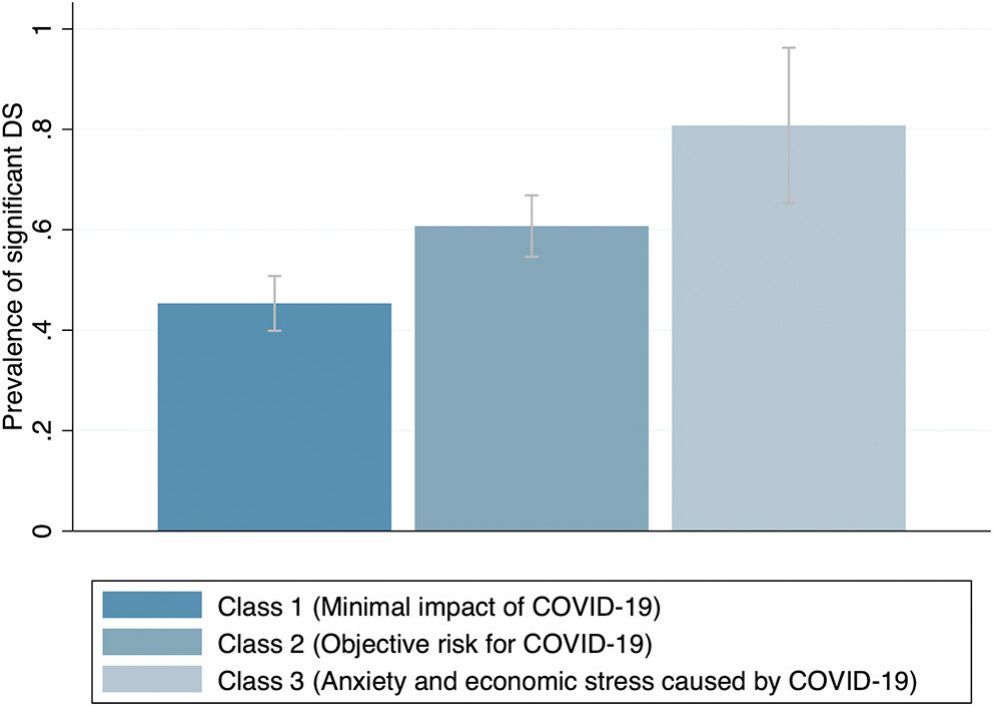
Prevalence of significant DS by COVID-19 effect class.

The prevalence of significant DS was 45, 60, and 80% for class one, two, and three, respectively (Figure 3). Besides education and state of residence, we did not observed differences in the distribution of demographic characteristics among classes. Regarding sexual behavior, those in class three reported less transactional sex compared with those in classes one and two (Supplementary Table 2).

In logistic regression models, latent class membership was associated with significant DS (Table 2, model 1). Compared with participants in class 1, those at risk for COVID-19 (class 2) were nearly two times more likely to have significant DS (OR 1.86, 95% CI 1.33–2.61); and those in class 3 were more than five times more likely to have significant DS (OR 5.06, 95% CI 1.86–13.76). The statistical significance and magnitude of these correlations were consistent after controlling for demographic characteristics (Table 2, model 2).

**Table 2:** Odds ratios from logistic regression models identifying associations of latent class assignment by posterior probability with significant depressive symptoms.

	**Model**	
**Variables**	**1**	**2**
**Class of COVID-19 effects**		
Class 1 (minimal impact of COVID-19)	Ref	Ref
Class 2 (objective risk for COVID-19)	1.86^***^	1.88^***^
	(1.33–2.61)	(1.32–2.66)
Class 3 (economic stress caused by COVID-19)	5.06^***^	4.98^***^
	(1.86–13.76)	(1.79–13.83)
**Demographics**		
Education level		0.84
		(0.51–1.36)
≥36 years		Ref
18–27 years		2.63^***^
		(1.57–4.43)
28–36 years		1.58^**^
		(1.03–2.43)
Lives in Mexico City (1 = yes)		1.67^***^
		(1.14–2.45)
Null social support		Ref
Some social support		0.56^*^
		(0.29–1.04)
High social support		0.48^**^
		(0.27–0.85)
Gender (1 = male)		0.54
		(0.18–1.65)
ImPrEP participant (1 = yes)		0.74
		(0.47–1.15)
Constant	0.83^*^	1.76
	(0.666–1.033)	(0.49–6.33)
Observations	595	595

*95% CI in parentheses;*

*** *p < 0.01,*

** *p <0.05,*

* *p <0.1*.

### Quantile Regression analysis

In Supplementary Table 3, we present the results of the quantile regression models to assess whether COVID-19 had a differential influence on DS across the distribution of the CESD-10 score. Column 1 shows the coefficients from the mean CESD-10 score, followed by the coefficients in the 25, 50, 75, and 90 quantiles of the distribution. Job loss because of COVID-19, higher perception of risk, and at least one medical risk factor for severe COVID-19 disease, were positively associated with the CESD-10 score in the 10, 25, 50, and 75 quantiles. However, the variables were not associated with DS among the most severely depressed participants (Q90), except for knowing someone diagnosed with COVID-19.

## Discussion

Our study aimed to assess the prevalence of significant depressive symptoms and their association with the COVID-19 pandemic among a sample of MSM and TGW in Mexico.

Significant DS were highly prevalent in our sample (53.3%), nearly seven times higher than men with a university degree or higher in ENSANUT 2018. Population-based mental health surveys have found consistently higher rates of major depression, anxiety, substance abuse, sexual and physical mistreatment, and suicidal behaviors in individuals disclosing same-sex sexual behavior or identifying as gay (22, 23).

However, our results support the hypothesis that the COVID-19 pandemic has increased the prevalence of DS substantially. DS were almost six times more prevalent in our study than in the baseline measurement in the same populations before the COVID-19 outbreak (51.9 vs. 8.6%). The prevalence of DS in our study was almost twice as high as the iPrEX OLE trial (51.9 vs. 28%) among MSM on PrEP in 2016 (14).

Furthermore, the prevalence of SD among the group of individuals in class 1 (minimally affected by COVID-19), was the lowest in our study −45.3%, compared with 60.7% in class 2 (objective risk), and 80.7% in class 3 (anxiety and economic stress). Our multivariate models showed that compared with those in class 1, participants in class 3 were almost five times more likely to present significant DS.

Our findings are consistent with previous studies showing that stressful situations like job loss are associated with an increased risk of DS and clinical depression (24). Job loss is a well-known risk factor for mental health conditions; however, our results add to recent literature documenting the high self-perceived risk of COVID-19 and contact with someone diagnosed with COVID-19 as risk factors. A recent study assessed the psychological effects of COVID-19 in the Chinese population and found that those with close contact with an individual with confirmed COVID-19 and those with a higher perceived risk of acquiring COVID-19 had a higher likelihood for depression (5).

We also observed a higher prevalence of significant DS among participants living in Mexico City, the state with the highest number of COVID-19 cases and deaths. Previous studies have shown that the prevalence of PTSD symptoms is higher in most affected communities by COVID-19. One study in China showed that 1 month after the outbreak, PTSD symptoms were present in 4.6% of mainland China population against 18.4% in provinces with a higher number of COVID-19 cases (25). In our study, DS were significantly more prevalent among people reporting less social support, consistent with previous studies showing that higher social support and better social connection quality lower the risk of DS (26). A study among young adults in the US found that high levels of loneliness were associated with clinical levels of depression, anxiety, and PTSD symptoms. The same study also found that having social support from family and friends was a significant predictor of low levels of depression and PTSD (27).

COVID-19-related variables were not significantly associated with the CESD-10 score in the higher quantile of the distribution (except for knowing someone diagnosed with COVID-19). Simultaneously, demographic characteristics such as age and state of residence were significant predictors of this quantile score, which might be associated with a specific profile of MSM and TGW, who suffered from severe depression before the outbreak.

One important implication of the high prevalence of DS is the potential impact of depression on other health outcomes of our participants. Mental health conditions, including depression, are known barriers to health care engagement and daily medication adherence. One study conducted among PrEP users in East Africa found that depressive symptoms were significantly associated with lower rates of PrEP adherence among participants (28).

Also, DS are related to increased HIV risk behaviors (multiple sexual partners, condomless sex), poor social support, and substance abuse (29). Among adults living with HIV, those with DS were 55% less likely to achieve optimal daily antiretroviral therapy (ART) adherence (30). Since MSM and TGW carry a disproportionate burden of the HIV epidemic (31), proper adherence to PrEP and other HIV prevention strategies is crucial to reduce the burden of HIV among these populations.

Our study is one of the firsts addressing the prevalence of DS and the association with COVID-19 related variables among HIV high-risk populations in Latin America. Although several studies have documented increases in the prevalence of depression, anxiety, and PTSD during the COVID-19 outbreak and lockdown, most of them were conducted in Asia and Europe and focused on the general population or health care workers (8). Because of that, the generalization of their results is limited for contexts like Latin America and to populations with very particular characteristics like MSM and TGW.

Our findings should be interpreted in light of several limitations of the study. First, baseline measurement of DS among the ImPrEP participants before social distancing measures is based on the SF-36 scale. However, previous studies have suggested that the SF-36 and CESD-10 depression scales measure similar constructs, and a strong correlation between both scores has been documented (32). Second, we did not collect information on how COVID-19 impacted participants' social support group. Third, those with complete information on all the variables included in our study were significantly more educated and younger than those with incomplete information (Supplementary Table 4). It is difficult to assess the direction of the bias introduced by this selection process. When comparing the ImPrEP participants' characteristics in our online survey with the ImPrEP's full sample, we observed that participants living in Mexico City are overrepresented (64% ImPrEP sample vs. 73% online survey). Since Mexico City is the most affected state in the country by COVID-19, this can explain the high prevalence of DS among the sample. Finally, our findings cannot be generalized because of the particular profile of our participants—-highly educated compared with the general population in Mexico.

As the COVID-19 epidemic continues to spread, we hope our findings will help inform strategies to minimize the impacts of COVID-19 on the mental health of MSM in Mexico and other places affected by the epidemic. PrEP and other HIV prevention programs across the globe will probably need to address this health problem among their users explicitly and include psychological interventions in their design.

## Conclusions

During the 1st month of the COVID-19 outbreak in Mexico, more than half of the MSM and TGW participants in this online survey had significant DS. Our findings emphasize the need for targeted psychological interventions for populations affected by COVID-19, particularly for individuals already at high risk for poor mental health outcomes. Mechanisms to better monitor and diagnose mental health disorders and improve access to psychological interventions to diminish or prevent future psychiatric morbidity among PrEP and non-PrEP users are necessary. Finally, additional research is needed to assess the potential impact of poor mental health on other outcomes, such as PrEP adherence, the adoption of HIV risk behaviors, and adherence to COVID-19 preventive measures.

## Data Availability Statement

The raw data supporting the conclusions of this article will be made available by the authors, without undue reservation.

## Ethics Statement

The study involving human participants was reviewed and approved by the Ethics Committee of the National Institute of Public Health (IRB number: CI-241-2020). Participants provided their written informed consent to participate in the study.

## Author Contributions

DC-G lead the analysis and wrote the first draft of the paper. HV, JG-C, AM-D, and IH-I collaborated in the design of instruments and reviewed the paper. JA conducted analyses and review the paper. IB reviewed the final version of the paper and contributed to interpretation of results. SB-A designed the original study and supervised the development of the manuscript. All authors contributed to the article and approved the submitted version.

## Conflict of Interest

The authors declare that the research was conducted in the absence of any commercial or financial relationships that could be construed as a potential conflict of interest.
