# Supplementary material for: Profiles of Depressive Symptoms Among Men Who Have Sex With Men and Transgender Women During the COVID-19 Outbreak in Mexico: A Latent Class Analysis
Source: Front Public Health. 2021 Jun 7;9:598921. doi: 10.3389/fpubh.2021.598921 (PMC8215204; doi:10.3389/fpubh.2021.598921)
Supplement: Supplementary file 1 [file Data_Sheet_1.docx]

Supplementary Material

Table S1. Comparison of demographic characteristics between ImPrEP and Online survey participants on PrEP

| **Variable** | **ImPrEP** | **Online survey on PrEP^a^** | **P value** |
| --- | --- | --- | --- |
| n(%) | 2,449 (100.0) | 675 (100.0) |  |
| Education^b^ |  |  |  |
| Less than university | 543 (22.2) | 124 (18.4) | 0.092 |
| University or higher | 1,906 (77.8) | 549 (81.6) |  |
| Gender |  |  |  |
| Men | 2,375 (96.9) | 654 (97.5) | 0.068 |
| Transgender women | 74 (3.02) | 17 (2.5) |  |
| Age (mean, sd) | 31.5 (7.9) | 31.8 (6.5) | 0.289 |
| State of residence |  |  |  |
| Mexico City | 1,550 (64.1) | 493 (73.0) | <0.001 |
| Lives in other state of Mexico | 868 (35.9) | 182 (27.0) |  |

^a^Includes participants with and without complete information who reported being enrolled in the ImPrEP study

^b^Sample sizes could vary due to missing data

Table S2. Characteristics of COVID-19 effect classes

|  | **Class** | | |  |
| --- | --- | --- | --- | --- |
| **Variable** | **Minimal impact of COVID-19 n=322** | **Objective risk for COVID-19 n=247** | **Anxiety and economic stress caused by COVID-19 n=26** | **P value** |
|  | **mean or % (SD)** | | |  |
| **Demographics** |  |  |  |  |
| Education |  |  |  |  |
| Less than university | 16.2 | 20.2 | 0.0 | 0.026 |
| University degree of higher | 83.9 | 79.8 | 100.0 |  |
| Age |  |  |  |  |
| 18-27 years | 25.5 | 25.9 | 15.4 | 0.595 |
| 28-36 years | 54.4 | 50.2 | 61.5 |  |
| >36 years | 20.2 | 23.9 | 23.1 |  |
| State of residence |  |  |  |  |
| Lives in Mexico City | 68.3 | 76.1 | 92.3 | 0.008 |
| Social support |  |  |  |  |
| Null social support | 12.1 | 10.1 | 15.4 | 0.736 |
| Some social support | 24.5 | 28.7 | 26.9 |  |
| High social support | 63.4 | 61.1 | 57.7 |  |
| Gender |  |  |  |  |
| Male | 97.2 | 96.8 | 100.0 | 0.638 |
| Transgender women | 2.8 | 3.2 | 0.0 |  |
| **Sexual behavior** |  |  |  |  |
| Sexual partners in last two weeks^a^ | 2.5 (2.9) | 2.3 (2.1) | 3.0 (3.8) | 0.744 |
| Type of sexual partner^a,b^ |  |  |  |  |
| Steady | 56.3 | 52.0 | 43.8 | 0.547 |
| Occassional | 65.6 | 65.1 | 70.6 | 0.905 |
| Transactional | 9.6 | 19.4 | 6.7 | 0.042 |
| Use of dating apps | 63.3 | 67.5 | 76.9 | 0.273 |

^a^ Among those who had sex (50.6% of the sample)

^b^ Non-mutually exclusive categories

Table S3. Quantile regression for factors associated with significant depressive symptoms among MSM and TGW

|  | **Model** | | | | | |
| --- | --- | --- | --- | --- | --- | --- |
| **Variables** | **mean** | **Q10** | **Q25** | **Q50** | **Q75** | **Q90** |
|  |  |  |  |  |  |  |
| Education level (1=university or higher) | -0.41 | 0.89 | -0.08 | -0.67 | -1.28 | 1.00 |
|  | (-1.76 - 0.93) | (-0.79 - 2.56) | (-1.35 - 1.19) | (-2.42 - 1.08) | (-3.50 - 0.94) | (-1.89 - 3.89) |
| >36 years | Ref | Ref | Ref | Ref | Ref | Ref |
| 18-27 years | 3.29*** | 1.22 | 2.58*** | 3.00*** | 4.06*** | 4.50*** |
|  | (1.87 - 4.70) | (-0.54 - 2.98) | (1.24 - 3.92) | (1.15 - 4.84) | (1.71 - 6.39) | (1.45 - 7.54) |
| 28-36 years | 1.62*** | 0.78 | 1.50*** | 1.00 | 1.89* | 1.50 |
|  | (0.42 - 2.81) | (-0.71 - 2.26) | (0.36 - 2.63) | (-0.55 - 2.55) | (-0.08 - 3.86) | (-1.07 - 4.07) |
| Lives in Mexico City (1=yes) | 1.97*** | 0.78 | 1.17** | 2.00*** | 2.17** | 2.50** |
|  | (0.91 - 3.03) | (-0.54 - 2.09) | (0.16 - 2.17) | (0.61 - 3.38) | (0.41 - 3.91) | (0.21 - 4.78) |
| Null social support | Ref | Ref | Ref | Ref | Ref | Ref |
| Some social support | -1.60* | 0.00 | -1.33 | -2.67** | -1.00 | 0.00 |
|  | (-3.28 - 0.08) | (-2.10 - 2.10) | (-2.93 - 0.26) | (-4.86 - -0.46) | (-3.78 - 1.78) | (-3.63 - 3.63) |
| High social support | -2.89*** | -1.11 | -2.08*** | -3.33*** | -3.17** | -2.50 |
|  | (-4.43 - -1.34) | (-3.04 - 0.82) | (-3.55 - -0.61) | (-5.35 - -1.31) | (-5.72 - -0.60) | (-5.83 - 0.83) |
| Gender (1=male) | -1.94 | 3.78** | 0.83 | -2.67 | -3.50 | -5.00 |
|  | (-4.84 - 0.96) | (0.15 - 7.40) | (-1.91 - 3.58) | (-6.45 - 1.12) | (-8.29 - 1.29) | (-11.25 - 1.25) |
| ImPrEP participant | -0.27 | 0.78 | -0.17 | -1.33 | 0.11 | -0.50 |
|  | (-1.52 - 0.98) | (-0.78 - 2.34) | (-1.35 - 1.02) | (-2.96 - 0.30) | (-1.95 - 2.18) | (-3.19 - 2.19) |
| Lost employment due to COVID-19 (1=yes) | 1.27** | 2.11*** | 1.25** | 1.33* | 1.28 | 1.50 |
|  | (0.14 - 2.39) | (0.70 - 3.51) | (0.18 - 2.31) | (-0.13 - 2.80) | (-0.58 - 3.13) | (-0.92 - 3.92) |
| Null perceived risk of COVID-19 | Ref | Ref | Ref | Ref | Ref | Ref |
| Some perceived risk of COVID-19 | 1.62** | 1.33 | 1.58** | 2.00** | 2.17* | -0.50 |
|  | (0.18 - 3.06) | (-0.46 - 3.12) | (0.21 - 2.94) | (0.12 - 3.87) | (-0.21 - 4.54) | (-3.59 - 2.59) |
| High perceived risk of COVID-19 | 3.20*** | 3.44*** | 4.08*** | 3.00*** | 4.11*** | 2.00 |
|  | (1.60 - 4.78) | (1.45 - 5.43) | (2.57 - 5.59) | (0.92 - 5.07) | (1.48 - 6.74) | (-1.42 - 5.42) |
| Has at least one risk factor for COVID-19 | 1.60*** | 1.00 | 1.25*** | 1.67*** | 1.89** | 0.50 |
|  | (0.63 - 2.55) | (-0.19 - 2.19) | (0.34 - 2.16) | (0.41 - 2.91) | (0.30 - 3.47) | (-1.56 - 2.56) |
| Knows someone with COVID-19 | 1.45*** | 0.33 | 1.33** | 1.67** | 2.44*** | 2.50** |
|  | (0.37 - 2.52) | (-1.00 - 1.67) | (0.31 - 2.35) | (0.26 - 3.06) | (0.66 - 4.22) | (0.18 - 4.81) |
| Had contact with someone with COVID-19 | -1.33 | 0.67 | 0.25 | -0.67 | -3.22 | -6.00 |
|  | (-4.24 - 1.57) | (-2.96 - 4.29) | (-2.50 - 3.00) | (-4.46 - 3.13) | (-8.03 - 1.58) | (-12.26 - 0.26) |
| Decreased sexual partners because COVID-19 | 0.06 | -0.44 | -0.17 | 0.17 | 0.42 | 0.25 |
|  | (-0.53 - 0.66) | (-1.18 - 0.29) | (-0.73 - 0.39) | (-0.61 - 0.94) | (-0.56 - 1.40) | (-1.03 - 1.53) |
| Constant | 9.05*** | -3.67 | 2.50 | 10.33*** | 12.61*** | 19.50*** |
|  | (5.42 - 12.67) | (-8.18 - 0.85) | (-0.93 - 5.93) | (5.60 - 15.06) | (6.62 - 18.60) | (11.69 - 27.30) |
| Observations | 595 | 595 | 595 | 595 | 595 | 595 |
| R-squared | 0.155 |  |  |  |  |  |
| 95% CI in parentheses |  |  |  |  |  |  |
| *** p<0.01, ** p<0.05, * p<0.1 |  |  |  |  |  |  |

Table S4. Comparison of demographic characteristics between online survey participants with complete and incomplete information

| **Variable** | **With complete information** | **With incomplete information ^a^** | **p value** |
| --- | --- | --- | --- |
| n (%) or mean (SD) | 595 (67.5) | 286 (32.5) |  |
| **Demographics** |  |  |  |
| Gender |  |  |  |
| Men | 578 (97.1) | 229 (95.8) | 0.328 |
| Transgender women | 17 (2.9) | 10 (4.2) |  |
| Education |  |  |  |
| Less than university | 102 (17.1) | 62 (24.8) | 0.010 |
| University degree of higher | 493 (82.9) | 188 (75.2) |  |
| Age | 31.9 (0.29) | 33.4 (0.54) | 0.007 |
| State of residence |  |  |  |
| Lives in Mexico City | 432 (72.6) | 180 (71.2) | 0.665 |
| Lives in other state of Mexico | 163 (27.4) | 73 (28.9) |  |
| Social support |  |  |  |
| Without any social support | 68 (11.4) | 34 (16.9) | 0.122 |
| At least one type of social support | 157 (26.4) | 47 (23.4) |  |
| Both types of social support | 370 (62.2) | 120 (59.7) |  |
| Depressive symptoms |  |  |  |
| With significant DS | 317 (53.3) | 24 (40.7) | 0.065 |
| Without significant DS | 278 (46.7) | 35 (59.3) |  |

^a^Sample sizes could vary due to missing data

Table S5. Latent class analysis model fit indices for two- through seven-class models of MSM and TGW

| **Number of latent classes** | **Degrees of freedom** | **AIC** | **BIC** | **Log-likelihood** |
| --- | --- | --- | --- | --- |
| 2 | 13 | 3214.50 | 3297.12 | -1594.25 |
| 3 | 20 | 3209.35 | 3271.55 | -1584.67 |
| 4 | 27 | 3214.97 | 3333.46 | -1580.49 |
| 5 | 33 | 3220.36 | 3365.18 | -1577.18 |
| 6 | 39 | 3231.42 | 3402.57 | -1576.71 |
| 7 | 43 | 3233.99 | 3422.70 | -1574.00 |
